# X Chromosome Reactivation Initiates in Nascent Primordial Germ Cells in Mice

**DOI:** 10.1371/journal.pgen.0030116

**Published:** 2007-07-27

**Authors:** Michihiko Sugimoto, Kuniya Abe

**Affiliations:** Technology and Development Team for Mammalian Cellular Dynamics, BioResource Center, RIKEN Tsukuba Institute, Tsukuba, Ibaraki, Japan; The Babraham Institute, United Kingdom

## Abstract

During primordial germ cell (PGC) development, epigenetic reprogramming events represented by X chromosome reactivation and erasure of genomic imprinting are known to occur. Although precise timing is not given, X reactivation is thought to take place over a short period of time just before initiation of meiosis. Here, we show that the cessation of *Xist* expression commences in nascent PGCs, and re-expression of some X-linked genes begins in newly formed PGCs. The X reactivation process was not complete in E14.5 PGCs, indicating that X reactivation in developing PGCs occurs over a prolonged period. These results set the reactivation timing much earlier than previously thought and suggest that X reactivation may involve slow passive steps.

## Author Summary

X chromosome inactivation is a mechanism to compensate gene dosage difference between XY males and XX females in mammals. During early embryogenesis, one of two X chromosomes in every female cell is inactivated, and the inactive X chromosome is stably inherited through cell divisions of somatic cells. Although precise timing is not given, the inactive X chromosome is known to be reactivated during germ cell development. It is generally believed that the dynamics of X chromosome activity is tightly correlated with major genomic reprogramming events occurring during mammalian development. Therefore, elucidation of the X reactivation kinetics is important for understanding the mechanism of X chromosome inactivation/reactivation processes and the epigenetic reprogramming processes as well. Here we investigated when X reactivation is initiated during development of female mouse germ cells. Contrary to the previous suggestions, X reactivation already begins in nascent primordial germ cells in female mice and proceeds gradually requiring a prolonged period. The activity status of the X chromosomes of germ cells appears to vary from cell-to-cell and from gene-to-gene during the reactivation processes. These results indicate that the X reactivation coincides with the formation of germ cells and suggest that this involves slow passive steps.

## Introduction

In mice, germ cell formation is first observed in postimplantation embryos; primordial germ cells (PGCs) appear at the base of allantois by embryonic day (E) 7.25 [1]. PGCs are unique compared to other cell types; extensive genomic reprogramming events such as erasure of genomic imprinting and reactivation of the inactive X chromosome take place in this cell type [2,3].

In female mammals, one of two X chromosomes in every cell is inactivated during early embryonic development to compensate gene dosage difference between XY males and XX females [4]. This phenomenon, X chromosome inactivation, represents one of the most remarkable examples of epigenetic gene regulation in mammals. In mouse, X inactivation randomly occurs in embryo proper, whereas the paternal X chromosome is preferentially inactivated in extraembryonic tissues [5,6]. X chromosome inactivation is regulated by a noncoding *Xist* RNA. It was shown that prior to X inactivation, *Xist* RNA is transcribed from both active X chromosomes. Upon differentiation, *Xist* RNA expression is upregulated on the future inactive X chromosome [7], and such *Xist* transcripts eventually coat the entire inactive X chromosome in differentiated cells [8–10]. Although its mechanistic role in gene silencing is not precisely known, the *Xist* RNA seems to be required for spreading the inactive state along the chromosome. The expression of *Xist* RNA is negatively regulated by *Tsix,* the antisense transcript of *Xist* [11,12].

The inactivated X chromosome is stably inherited through cell divisions of somatic cells. However, it is known that the two X chromosomes are active in oocytes [13–15], indicating that the inactive X chromosome must be reactivated during germ cell development. Previously, it was thought that X reactivation occurs in PGCs following entry into the gonads just before the initiation of meiosis [16–19] and that the reactivation coincides with rapid DNA demethylation of PGC genome, which is completed in one day of development [2]. Recent studies have shown that X reactivation also occurs in preimplantation development. The paternal X chromosome is inactivated at cleavage stage embryos of female mouse [20–22]. The inactive paternal X chromosome is reactivated in epiblast cells of peri-implantation embryos before the onset of the random X inactivation [21,23]. This reactivation proceeds rapidly and appears to be completed in ∼24 h [21]. Reactivation just after fertilization has also been suggested (reviewed in [24]).

These findings indicate that reactivation of the inactive X chromosome occurs at least twice during mammalian development, once in the epiblast cell lineage at the peri-implantation stage and once in the PGCs at the midgestation stage, and that the reactivation of the inactive X chromosome appears to be tightly correlated with major genomic reprogramming events occurring during mammalian development [25]. Therefore, elucidation of the X reactivation kinetics is important for understanding the mechanism of X chromosome inactivation/reactivation processes and the epigenetic reprogramming processes as well. While epigenetic dynamics of X chromosome inactivation and reactivation in pre- and peri-implantation stage embryos have been studied in detail [20–22], the timing of X reactivation in developing female PGCs has not been clearly defined. The results presented in the previous reports were somewhat contradictory, possibly because of the technical difficulties in analyzing the activity of X chromosomes in developing germ cells [17–19,26].

Here, we devised novel sensitive assays to determine the timing of X reactivation and demonstrated that, contrary to the previous suggestions, X chromosome reactivation has already been initiated in newly formed female germ cells as early as E7.0 and that the reactivation requires a prolonged period (≥7 d), suggesting that the reactivation may involve slow passive processes.

## Results

### Whole Mount RNA FISH Analysis of *Xist* Accumulation in Developing PGCs

**Figure 1:**
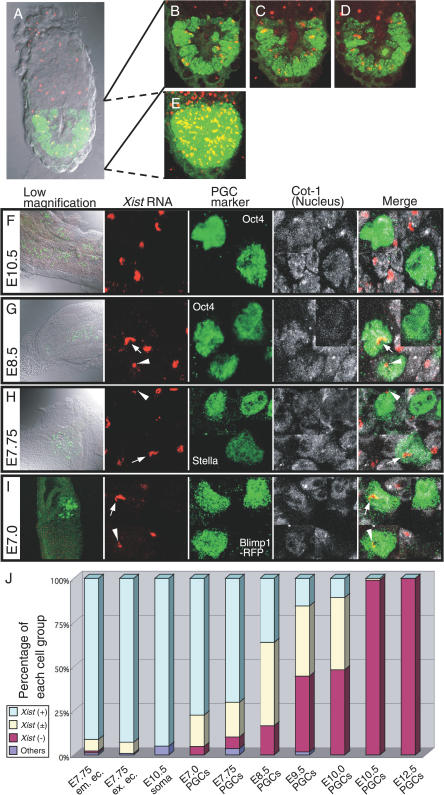
Results of Whole-Mount RNA FISH with *Xist* Probe (A–E) An E5.5 embryo-expressing methyl-CpG binding domain (MBD)-GFP fusion protein (green) under the control of *Oct4* promoter is shown (S. Kobayakawa and K. Abe, unpublished data). This transgene was used to visualize the nucleus of epiblast. (A) Shown is a single optical section merged with DIC image. Images in panels (B–D) were obtained by projecting ten optical slices at different depths of the specimen shown in (A). (E) A projection image from all 230 optical slices is presented. Red is *Xist* RNA signal, green is GFP fluorescence. (F–J) Whole-mount RNA FISH used *Xist* (red) and Cot-1 (white) probes combined with immunofluorescence for PGC markers (green) with antibodies against Oct4 (F, G), Stella (H), and RFP (I). Images in each panel were obtained by projecting ten optical slices. *Xist* RNA did not accumulate in the nuclei of E10.5 PGCs (F). *Xist* (+) PGCs (arrow), *Xist* (±) PGCs (arrowhead), and also *Xist* (−) cells, were present at E8.5 (G), E7.75 (H), and E7.0 (I). (J) Percentages of *Xist* (+), *Xist* (±), and *Xist* (−) cells in the cell populations tested. As controls, the percentages of cells with each *Xist* signal pattern are indicated for the embryonic ectoderm (em. ec.) and extraembryonic ectoderm (ex. ec.) in E7.75 embryos and for the somatic cells surrounding the PGCs in E10.5 embryos. Cells with scattered *Xist* signals in their nuclei are classified as “others.”

The accumulation of *Xist* RNA is a hallmark of the inactive X chromosome [8–10]. *Xist* expression is usually detected by RNA fluorescence in situ hybridization (FISH) analysis of dissociated cells adhered to slide glasses. As PGCs comprise a small cell population surrounded by somatic cells throughout the embryogenesis, dissociation of embryos during sample preparation would lead to loss of PGCs or bring a contamination of somatic cells into the specimens. To avoid these difficulties, an RNA FISH method that detects RNA expression in intact embryos is required. For this purpose, we devised a novel whole-mount RNA FISH method that allows the sensitive detection of *Xist* RNA without sacrificing the integrity of the embryonic structure (Figure 1A–1E). Using an *Xist* probe and an antibody against PGC-specific markers, Oct4, we were able to accurately locate PGCs in embryos and assess *Xist* expression. A Cot-1 probe, which hybridizes to nascent RNA, was used to visualize the nuclei of specimens. Although the Cot-1 signal is eliminated from the inactive X chromosome [20], it was not clear whether the “Cot-1 hole” was present on the inactive X chromosome using our whole-mount RNA FISH method (Figures 1F–1I and S1). We first analyzed the genital ridges isolated from female embryos at E12.5, at which time the PGCs were about to enter meiosis. At this stage, all the PGCs were completely negative for *Xist,* whereas the somatic cells surrounding the PGCs had a large and strong *Xist* signal, indicating the presence of an inactive X chromosome (Figure S1; Table 1). At E10.5, when the PGCs began to colonize the genital ridges [3], almost all the PGCs were already *Xist*-negative (Figure 1F; Table 1), suggesting that X reactivation occurs earlier than previously thought.

**Table 1:**
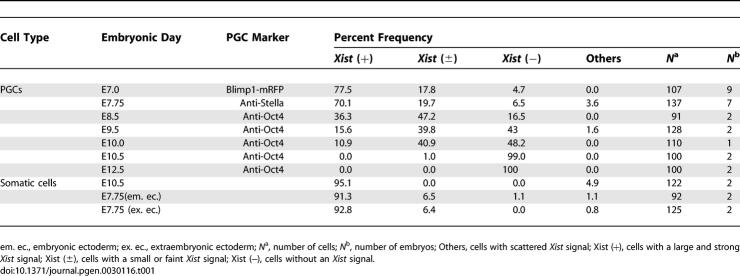
Distribution of PGCs with Each Type of *Xist* Signal

### PGCs Are Heterogeneous in Terms of Their *Xist* Expression Patterns during E7.75–E10.0

*Xist*-negative PGCs were found even at E8.5, comprising 16.5% of the total cells examined. The *Xist*-negative PGCs increased to 48.2% at E10.0. PGCs with strong *Xist* expression comprised 36.3% of total PGCs at E8.5 and 10.9% at E10.0. Most of the remaining cells displayed an *Xist* signal that was smaller than those of the somatic cells (Figure 1G), and a small number of cells had a large but very faint *Xist* signal. We hereafter describe those cells showing a small or faint *Xist* signal as *Xist* (±) cells. Cells with a large and strong *Xist* signal are described as *Xist* (+), and cells with no *Xist* signal as *Xist* (−). To investigate whether the cessation of *Xist* expression is initiated at an even earlier stage, we examined PGCs at E7.75, identified by an antibody against one of the earliest PGC markers, Stella (also known as PGC7) [27,28]. About 70% of Stella-positive PGCs were *Xist* (+), whereas ∼20% were *Xist* (±) (Figure 1H; Table 1). Unexpectedly, *Xist* (−) PGCs already existed at this early stage, although their frequency was lower than that of *Xist* (−) PGCs in later stages, but higher than that of somatic cells (Table 1).

### The Cessation of *Xist* Expression Already Commences in Nascent PGCs (or PGC Precursor Cells) at E7.0

PGCs are first identified in mice as a cluster of alkaline phosphatase-positive cells at E7.25 [3]. It was recently shown that *Blimp1* expression marks nascent PGCs as well as precursor of PGCs [29,30]. To explore the X inactivation status of the earliest PGCs (or their precursors), we generated bacterial artificial chromosome (BAC) transgenic mouse lines in which the gene for monomeric red fluorescent protein (mRFP) [31] was inserted into the *Blimp1* locus (Figure S2). mRFP expression was detected at the posterior end of the embryonic ectoderm and visceral endoderm in E7.0 embryos, consistent with endogenous *Blimp1* expression (Figure S2) [29]. Because the mRFP faded from the visceral endoderm after the embryos were processed for the whole-mount RNA FISH experiment, only nascent PGCs were clearly visualized (Figure 1I). As in the case of E7.75 PGCs, a minor but significant proportion of mRFP-positive cells (4.7%) were *Xist* (−) (Figure 1I; Table 1). About 18% of the mRFP-positive cells were *Xist* (±), whereas the rest of the cells were *Xist* (+). In contrast, all the mRFP-negative cells, i.e., the somatic cells, observed at the same stage were *Xist* (+), although about 4% of them were *Xist* (±) (unpublished data). Interestingly, *Xist* (±) PGCs continued to be present until their arrival at the genital ridges (maximum 47.2% at E8.5) (Figure 1J; Table 1). It is thus likely that X reactivation was in progress in those cells.

### Biallelic Expression of X-Linked Genes Is Detected in E7.75 PGCs

**Figure 2:**
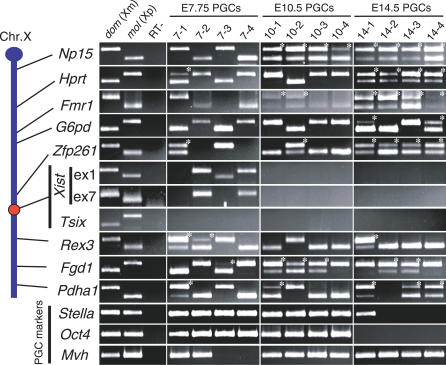
Biallelic Expression of X-Linked Genes in PGCs Revealed by Single-Cell RT-PCR Some X-linked genes are already expressed biallelically (asterisks) in some PGCs at E7.75, but that not all X-linked genes are biallelically expressed, even at E14.5. A total of four PGCs at each stage are indicated. Biallelic expression is marked with asterisks. The relative positions of the X-linked genes are roughly indicated.

**Figure 3:**
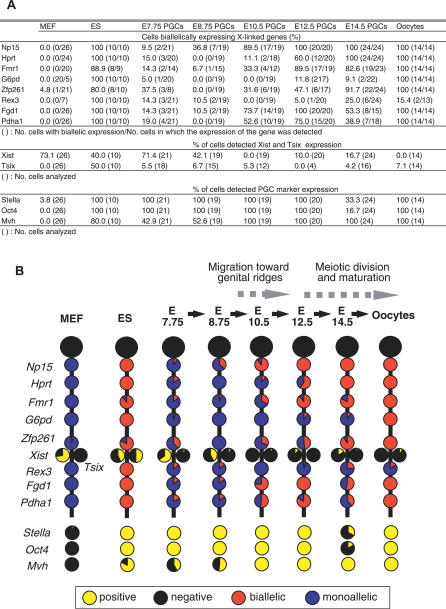
Expression Patterns in PGCs at Each Stage (A) Ratio of cells biallelically expressing X-linked genes is presented. (B) A schematic shows the transition of gene expression patterns during PGC development. Each circle graph indicates the ratio of cells that are positive (yellow) and negative (black) for each gene, and biallelically (red) and monoallelically (blue) expressed in cells positive for each gene.

To examine the X reactivation process from a different perspective, we next performed single-cell reverse-transcriptase PCR (RT-PCR) analysis of X-linked gene expression in individual PGCs. We used an Oct4-green fluorescent protein (GFP) transgenic mouse line in which the PGCs were specifically marked by reporter expression (Figure S3) [32,33] and crossed transgenic female mice with male MSM/Ms mice, an inbred mouse strain derived from *Mus musculus molossinus* subspecies [34]. Because the genetic background of the Oct4-GFP mice was *M. m. domesticus* type, SNPs were readily detectable between the Oct4-GFP and MSM/Ms strains and were used to detect the allele-specific expression of each X-linked gene in the hybrids. GFP-tagged PGCs were picked individually from hybrid female embryos and subjected to the RT-PCR analysis. We analyzed the expression of ten X-linked genes, including *Xist* and *Tsix,* and three PGC markers *(Stella*, *Oct4,* and *Mvh),* as shown in Figure 2. *Xist* expression was examined using two pairs of primers mapped to exon 1 and exon 7 of the *Xist* gene. Mouse embryonic fibroblasts (MEFs) isolated from (BDF1 × MSM/Ms) F1 female embryos and hybrid female embryonic stem (ES) cells derived from (Oct4-GFP × MSM/Ms) F1 blastocysts were used as controls (Figure S4). In our hands, *Xist* expression was detected in about 73% of single MEFs (Figure S4; Table S1). In all MEFs analyzed, most of the X-linked genes were monoallelically expressed from either the maternal Oct4-GFP or the paternal MSM/Ms X chromosome, and no *Tsix* expression was detected. The biallelic expression of *Zfp261* was detected in only one of the 26 MEF samples. It is known that both of the X chromosomes in female ES cells are active [6]. Our single-cell RT-PCR analyses successfully detected biallelic expression of all the X-linked genes tested in ES cells. Results obtained with MEFs and ES cells proved that our procedure could effectively distinguish mono- and biallelic expression of the X-linked genes in a single cell (Figure S4). In E7.75 PGCs, the expression of *Xist* was detected in 15 of 21 cells (71.4%) at a rate similar to that observed in MEFs (Figure 3A; Table S1). Most of the X-linked genes were monoallelically expressed in 13 of 21 samples. Interestingly, however, the biallelic expression of at least one gene was observed in eight of 21 PGC samples (Figures 2 and 3A; Table S1). In particular, in one PGC, all of the X-linked genes showed biallelic expression (Table S1). These findings demonstrate that one of two X chromosomes was inactivated in most of these early PGCs, which in turn suggests that the derepression of some of the X-linked genes begins in the newly formed PGCs or in even earlier PGC precursors. *Tsix,* the antisense transcript from the *Xist* locus, is thought to be a regulatory factor for X inactivation that acts by repressing *Xist* transcription [11,12]. *Tsix* expression was, however, barely detectable in early PGCs, and this trend persisted into later stages.

### Reactivation of X-Linked Genes Is Not Complete after the Initiation of Meiosis

At E8.75, *Xist*-positive PGCs decreased to 42.1% (Figure 3A; Table S1), whereas the derepression of the X-linked genes did not proceed significantly from the E7.75 stage. At E10.5, *Xist* expression was no longer observed in PGCs, consistent with the RNA FISH results. Derepression of three X-linked genes, i.e., *Np15*, *Fgd1,* and *Pdha1*, was obvious in more than half the samples (Figure 3A; Table S1). At E12.5, the number of biallelically expressed genes increased even further; five of eight X-linked genes (*Np15*, *Hprt*, *Fmr1*, *Fgd1,* and *Pdha1*) were expressed biallelically in more than 50% of PGCs. However, there was no single cell in which X reactivation had been completed at this stage (Figures 3A and S4; Table S1). As shown in Figure 3, derepression of the X-linked genes did not appear to proceed linearly, probably due to the limited number of PGCs assayed. However, most of the X-linked genes tested showed gradual reactivation in the course of PGC development (Figure 3B).

The prevailing view suggests that X reactivation begins soon after the entry of PGCs into the genital ridge and before the initiation of meiosis [16–19]. However, even at E14.5, when most of the female PGCs are in meiotic prophase [3], derepression appeared to be incomplete. Two genes, *Np15* and *Hprt,* were reactivated in all the samples, but other genes showed variable results (Figure 3B; Table S1). In particular, *G6pd* and *Rex3* tended to show monoallelic expression. Because all the genes except *Rex3* were completely reactivated in oocytes, E14.5 PGCs are probably still in the process of X reactivation.

Unexpectedly, we found that many single PGCs at E14.5 expressed one gene from the paternal X chromosome and the other from the maternal X chromosome. For example, as shown in lane 14–2 of Figure 2, in this particular cell, *G6pd* showed monoallelic expression from the maternal *domesticus* type X chromosome, whereas *Rex3* was expressed from the paternal *molossinus* allele. Such “mosaic” patterns of expression were frequently (11/24) observed in E14.5 PGCs but rare in earlier PGCs. Only one each of PGC at E7.75 and E8.75 showed a mosaic pattern (Table S1). It is likely, therefore, that this is a phenomenon specific to E14.5 PGCs or to PGCs undergoing meiosis.

## Discussion

Here, we showed that the cessation of *Xist* RNA transcription had already been initiated in newly formed PGCs, and some X-linked genes were actually reactivated in these nascent PGCs. Furthermore, *Xist* accumulation was completely undetectable at E10.5, more than two days before the initiation of meiosis in female PGCs. However, the reactivation of X-linked genes was not complete even at E14.5, when meiotic division is initiated in most of female PGCs.

Despite their biological importance, the timing of both the inactivation and reactivation of the X chromosome has not been precisely determined in PGC. In previous studies of X reactivation, the results were based on the expression of protein products, which lags behind dynamic changes in RNA expression. Somatic contamination of germ cell samples might have disturbed previous studies. In contrast, we assessed the inactivation status in PGCs using whole-mount RNA FISH and measured X chromosome activity directly by detecting the allelic transcripts of ten X-linked genes in single PGCs. The use of multiple appropriate markers for each stage of PGC development further increased the validity of our assay.

Among the earliest PGCs marked by *Blimp1* expression, there were some cells showing no *Xist* expression, and hence undergoing X reactivation. This may imply delayed X inactivation in the germ line as suggested previously by Tam et al. [18], who assessed the X inactivation and reactivation status by examining the X-linked *lacZ* transgene expression. However, even at E7.0 a large number of cells (77.5%) possessed *Xist* paint signals, indicating that most of the PGCs and/or their precursor cells are not exempt from X inactivation. Because the numbers of such *Xist* (+) cells decreased and *Xist* (−) cells increased progressively in number, the simplest interpretation is that while every epiblast cell in the early egg cylinder undergoes X inactivation once, PGC precursor cells in the epiblast are somehow susceptible to reactivation. Thus, X reactivation is likely to occur slightly before the advent of PGC. It is tempting to speculate that the initiation of X reactivation coincides with the determination of the germ-cell lineage.

We found that a significant number of X-linked genes showed monoallelic expression even though *Xist* painting was no longer observed. Similar observation made by Csankovszki et al. [35] suggests that continued *Xist* expression is not essential for maintenance of the inactive state after establishment of the X inactivation in vitro. Here, we showed that *Xist* expression and accumulation were hardly detected in E10.5 PGCs, but several X-linked genes were still monoallelically expressed even at E14.5. To our knowledge, these results are the first to demonstrate that the maintenance of X inactivation does not require *Xist* RNA accumulation on the inactive X chromosome in normal embryonic development in vivo.

Furthermore, we observed “mosaic” patterns of mono- and biallelic expression of X-linked genes in E14.5 PGCs in which no *Xist* signals were detected as described above. This phenomenon could be explained by very low expression levels of these genes at this stage, causing biased PCR amplification resulting in “pseudo” monoallelic expression. However, our microarray data (N. Mise and K. Abe, unpublished data) showed that steady state mRNA levels of these genes were high (and constant) at both E12.5 and E14.5 stages. Therefore, it is likely that the mosaic expression pattern may be caused by intermingling of inactive and active chromosomal regions through meiotic recombination. Further investigations on this mosaicism would provide insight into roles of “chromatin environment” in establishment and maintenance of X inactivation status.

Another important finding presented here is that X reactivation is not completed within a narrow window of time during development. Genome reprogramming events in PGCs represented by imprinting erasure and X reactivation are thought to take place over a short period of time, perhaps during the transition from pregonadal to gonadal PGCs [2]. Our results demonstrate that X reactivation proceeds gradually in a stepwise fashion, requiring a relatively long time (≥7 d). As the first step, the cessation of *Xist* occurred, and reactivation of a few X-linked genes already commenced during E7.0–E10.5. By E10.5, *Xist* accumulation on the inactive X chromosome has almost completely gone. After E10.5, reactivation of X-linked genes was accelerated significantly, and most of the X-linked genes tested showed biallelic expression in the next two days. As this second phase of reactivation is more prominent than the first one, previous studies might have overlooked the early commencement of the X reactivation. However, the X reactivation in fact has initiated as soon as PGC differentiation occurs, and this fact may change a general view on X inactivation/reactivation and genomic reprogramming phenomena in the germ cell lineage. During PGC development, their number increases from 50 to 25,000 [36], involving more than nine cell divisions. The activity status of the X chromosomes of PGCs during this period appears to vary from cell-to-cell and from gene-to-gene. There are certain tendencies for some genes to be reactivated earlier than other genes, and one could also argue that genes distant from the *Xist* locus tend to be reactivated earlier than genes close to the *Xist*. Further extended analyses on expression of other X-linked genes and their surrounding genomic sequences will be needed to make definitive statements on this point.

The present study suggests that X reactivation in PGCs is not involved in active epigenetic remodeling, but may instead involve slow passive steps that require many cell divisions. In contrast, X reactivation events observed at peri-implantation stages (and zygotic gene activation) are completed in a much shorter period of time [21–23]. Kinetics of X reactivation in PGCs is rather similar to that of the reversal of X inactivation induced by cell fusion between an embryonal carcinoma cell and female somatic cell [37], for which four to five cell divisions are required [38]. These differences in kinetics of each reactivation process seem to reflect differences in mode of epigenetic regulation operating in random and imprinted X inactivation.

For maintenance of random X inactivation, methylation of CpG sites at the 5′ region of X-linked genes is important (reviewed in [6]). In a mutant deficient for maintenance DNA methyltransferase *(Dnmt1),* random X inactivation in the embryonic lineage is unstable, whereas the imprinted X inactivation in the extraembryonic lineage was unaffected [39], suggesting differences in the epigenetic state of the X chromosome in the two lineages. It is generally thought that global DNA methylation level is relatively low in the extraembryonic lineage and that imprinted X inactivation in this lineage is less stable compared to random X inactivation in embryonic lineage. In the extraembryonic lineage, therefore, the epigenetic gene-silencing mechanism may not rely on DNA methylation [39,40], and erasure of the repressive state may be more readily accomplished. In contrast, DNA methylation is apparently important for establishment and maintenance of random X inactivation in embryo proper [39], and reactivation of the DNA methylation-dependent inactive state may require a longer period. Seki et al. [41] reported that global DNA methylation is substantially removed from migrating PGCs at around E8.0, and we also found that expression of all the DNA methyltransferases is hardly detected at an even earlier stage (S. Kobayakawa and K. Abe, unpublished data), suggesting that genome-wide erasure of DNA methylation commences from a very early stage of PGC development. Seki et al. [41] also demonstrated that global DNA methylation was further reduced between the stages E9.5 and E12.5. Such two-step reduction of global DNA methylation approximately coincides with the stepwise processes of X reactivation described here. Therefore, the unique epigenetic properties of the PGC genome likely constitute a foundation for the prolonged reactivation process.

As described, X reactivation may be a consequence of genome-wide epigenomic remodeling in PGCs, but the reactivation should have its own biological significance. One obvious reason is to avoid producing “inactive X–Y” offspring, in which no active X chromosome exists. On the other hand, female mice carrying only one X chromosome, X0 mice, are fertile, and in X0 female embryos PGCs have a single active X chromosome throughout their development. During maturation, some X0 oocytes release a first polar body containing the X chromosome and become mature oocytes without the X chromosome. When these “0-oocytes” fertilize with “X-sperm,” they develop as phenotypically normal female mice. Therefore, twice the amount of transcripts from the X chromosome is not essential for female germ cell development.

We think that the presence of two active (or euchromatic) X chromosomes may be needed for successful meiotic recombination. The inactive X chromosome is highly heterochromatic (reviewed in [6]), and heterochromatic chromosomal regions are generally underrepresented in the synaptonemal complex [42]. Pairing of active and inactive X is thus difficult to achieve, resulting in strong recombination suppression. Therefore, X reactivation in germ cells may have significance for appropriate segregation of X chromosome into mature oocytes.

In conclusion, we made an unexpected finding that initiation of X reactivation coincides with onset of PGC formation. Germ cell specification appears to be associated with genome-wide epigenetic reprogramming [41], raising the issue of how chromosome-wide X reactivation and genome-wide epigenetic changes are related. The kinetics of the X reactivation indicates that this process requires a prolonged period, suggesting that passive mechanisms may be involved. We found negligible *Tsix* expression in PGCs during the reactivation process. Recently, it was shown that CpG methylation of the *Xist* promoter region is mediated by *Tsix* transcription itself [7,43]. However, results presented in this study suggest that repression of *Xist* transcription during the reactivation process in PGCs is probably not mediated by *Tsix* transcription. Therefore, X reactivation is not exactly a reversal process of X inactivation. Apparently X reactivation process holds many important, unanswered questions, and we believe that investigation on this process will lead to better understandings of mechanisms of X inactivation, genomic reprogramming, and germ–soma differentiation in mammals.

## Materials and Methods

### Mice.

To create a transgenic construct for mRFP under the control of the *Blimp1* regulatory elements, we used a highly efficient Escherichia coli-based chromosome engineering system [44]. Homologous sequences for *Blimp1* were introduced to both ends of the mRFP-BGHpA-FRT-Neo-FRT sequence by PCR. Primers used for this amplification were 5′-CTAGCTCCGGCTCCGTGAAGTTTCAAGGACTGGCAGAGACTGGGATCATGATGGCCTCCTCCGAGGACGT-3′ and 5′-AACTCGGCCTCTGTCCACAAAGTCATATCAGCGTCCTCCATGTCCATTTTTGTGGAATTGTGAGCGGATA-3′. The resulting amplified fragment was recombined immediately after the methionine of exon 3 in the *Blimp1* gene in a 203-kb BAC, RP23-1D12, purchased from BACPAC Resources Center (http://bacpac.chori.org), carrying the 146-kb upstream region from the transcription start site and the 35-kb downstream region from the 3′ end of the 3′ untranslated region of *Blimp1* (Figure S2). The mRFP cDNA clone was kindly provided by Dr. Roger Tsien (Howard Hughes Medical Institute at the University of California, San Diego, California, United States, http://www.tsienlab.ucsd.edu).

The Oct4-GFP transgenic mouse line has been described previously [33] and is available from the RIKEN BioResource Center (http://www.brc.riken.jp/inf/en) as the TgN(deGFP)18Imeg strain. These mice carry the GFP sequence driven by an 18-kb *Oct4* genomic fragment with deletion of the proximal enhancer [32].

The MSM/Ms mouse strain was derived from *M. m. molossinus* mice [34]. Because the BDF1 (*M. m. domesticus*) and MSM/Ms mice are quite divergent, this F1 hybrid is rich in SNPs.

All methods were approved by the Institutional Animal Experiment Committee of RIKEN BioResource Center.

### Whole-mount RNA FISH.

A probe to detect *Xist* RNA was prepared by nick translation with Cy3-dCTP (GE Healthcare, http://www.gehealthcare.com) or SpectrumGreen-dUTP (Vysis, http://www.vysis.com) from an equimolar mixture of a series of *Xist* cDNA clones encompassing exons 1–7 (kindly supplied by Dr. Takashi Sado) [12]. Cot-1 DNA was also labeled by nick translation with Cy5-dCTP (GE Healthcare).

Isolated embryos were incubated in 0.5% Triton X-100 in PBS for 3–5 min on ice and fixed with 4% paraformaldehyde in PBS for 10 min at room temperature. Staging of the embryos was according to Downs and Davies [45] and Kaufman [46]: Our E7.75 embryos corresponded to either late bud (LB) or early headfold (EHF) stages [45]; E8.5 embryos corresponded to Theiler's stage 12, and E9.5 embryos were at Theiler's stage 14 [46]. Appearances of Oct4-GFP-positive PGCs were shown in Figure S3. Hybridization was carried out at 37 °C overnight. Following stringent washing, the embryos were incubated for 1 h at room temperature with primary antibody diluted with blocking buffer (1% BSA, 0.1% Tween 20, and 4× SSC): either 1:200 anti-Oct4 (sc-8628, Santa Cruz Biotechnology, http://www.scbt.com), 1:1,000 anti-Stella (kindly supplied by Dr. Toru Nakano) [28], or 1:500 anti-RFP (MBL, http://www.mbl.co.jp/e). The embryos were incubated in secondary antibody diluted in blocking buffer (1:500 Alexa-Fluor-488-conjugated rabbit antigoat IgG, Alexa-Fluor-488-conjugated goat antirabbit IgG, or Alexa-Fluor-555-conjugated goat antirabbit IgG, Invitrogen, http://www.invitrogen.com) for 45 min at room temperature. Because of the Triton X-100 treatment before fixation, mRFP expression in visceral endoderm was faded as shown in Figure 1I. Double staining with another PGC marker confirmed that mRFP expression was specific to PGCs. The embryos were mounted in 90% glycerol, 0.1× PBS, and 1% Dabco (Sigma-Aldrich, http://www.sigmaaldrich.com). Fluorescent images were taken with an LSM510 meta confocal laser scanning microscope (Zeiss, http://www.zeiss.com).

### Single-cell RT-PCR.

Embryos from Oct4-GFP × MSM/Ms matings were dissected at each stage. Following trypsinization, single GFP-positive cells were picked and stored at −80 °C until use. As control cells, single embryonic fibroblast cells prepared from E12.5 female embryos derived from BDF1 × MSM/Ms matings and single (Oct4-GFP × MSM/Ms) F1 hybrid ES cells, which were established previously in our laboratory (N. Mise and K. Abe, unpublished data), were used. The sexes of the E12.5 and E14.5 embryos were determined by the morphology of the genital ridges and by PCR in the earlier stage embryos. A single primer pair (5′-TGGATGGTGTGGCCAATG-3′ and 5′-CACCTGCACGTTGCCCTT-3′) amplifies both the X-linked *Ube1x* and Y-linked *Ube1y* sequences but yields products of different sizes (*Ube1x*, 252 bp and *Ube1y*, 334 bp).

Single cells were incubated in reverse transcription buffer supplemented with 0.1% NP-40 and 0.5 U of RQ1 RNase-free DNase (Promega, http://www.promega.com) for 15 min at 37 °C, for 3 min at 75 °C, and for 5 min on ice. Reverse transcription was carried out by adding 0.5 μl of 0.5 μg/μl oligo dT_18_ primer, 0.5 μl of 10 mM dNTP mix (Invitrogen), and 0.5 μl of 200 U/μl SuperScript III reverse transcriptase (Invitrogen). This was followed by incubation at 50 °C for 1 h. The reactions were incubated at 37 °C for 15 min with 1 U of RNase H (Invitrogen). We carried out two rounds of PCR amplification of the cDNA to detect 11 sequences of ten X-linked genes (exons 1 and 7 of *Xist, Tsix, Np15, Hprt, Fmr1, G6pd, Zfp261, Rex3, Fgd1,* and *Pdha1*) and the sequences of three PGC markers *(Stella, Oct4,* and *Mvh)* in single cells. The X-linked genes, apart from *Xist* and *Tsix,* were selected because they showed relatively high expression in PGCs according to our microarray analysis (unpublished data). We designed these primer sequences carefully to avoid amplifications of pseudogenes and to include SNPs in the amplicons for distinction of BDF1 and MSM/Ms alleles. For the first PCR, we used a mixture of all the primers listed in Table S2 to amplify all of the sequences in 100-μl reactions. Aliquots (0.5 μl) of the first PCR products were used as templates for the second PCR in 20-μl reactions. Each primer pair listed in Table S2 was used to amplify specific sequences in each single-cell-derived sample. For each series of experiment, we included negative controls; a single cell was processed in the same way as the experimental group except for the addition of reverse transcriptase. We had no amplification at all from negative controls. The second PCR products were digested with the appropriate restriction enzymes (Table S2).

## Supporting Information

Figure S1 Whole-Mount RNA FISH with *Xist* Probe in E12.5 Female PGCs *Xist* RNA signal is red, and Cot-1 signal is white. Oct4 immunofluorescence (green) was used to identify PGCs. *Xist* signal was not detected in any Oct4-positive cells at this stage.
(195 KB PDF)

Figure S2 Generation of the Blimp1-mRFP Transgenic Mouse Line (A) Transgene construct expressing mRFP under the control of *Blimp1* regulatory elements is shown (see Materials and Methods).
(B) Transgene expression was observed under a fluorescence stereomicroscope. mRFP fluorescence was detected as a cluster of cells in the extraembryonic mesoderm (arrowhead) and in the visceral endoderm.
(173 KB PDF)

Figure S3 Oct4-GFP Expression Patterns during Embryogenesis Tissues containing PGCs were isolated at E14.5 (A), E12.5 (C), E10.5 (E), E8.75 (G), and E7.75 (I). PGCs were identified as GFP-positive cells (B, D, F, H, and J) and picked manually (K, L).
(311 KB PDF)

Figure S4 Single-Cell RT-PCR Results of E8.75 PGCs, E12.5 PGCs, Oocytes, MEFs, and ES Cells E8.75 and E12.5 PGCs were isolated from hybrid embryos generated from Oct4-GFP × MSM/Ms matings, and oocytes were from superovulated hybrid females. MEFs and ES cells were used as controls. Asterisks indicate biallelic expression.
(466 KB PDF)

Table S1 Detailed Expression Patterns of X-Linked Genes and PGC Marker Genes in Single Cells (38 KB PDF)

Table S2 List of Primer Pairs and Restriction Enzymes Used in Single-Cell RT-PCR Analyses (16 KB PDF)

## Abbreviations

E: embryonic day
ES: embryonic stem
FISH: fluorescence in situ hybridization
GFP: green fluorescent protein
MEF: mouse embryonic fibroblast
mRFP: monomeric red fluorescent protein
PGC: primordial germ cell
RT-PCR: reverse-transcriptase PCR

## References

[pgen-0030116-b001] Ginsburg M, Snow MHL, McLaren A (1990). Primordial germ cells in the mouse embryo during gastrulation. Development.

[pgen-0030116-b002] Hajkova P, Erhardt S, Lane N, Haaf T, El-Maarri O (2002). Epigenetic reprogramming in mouse primordial germ cells. Mech Dev.

[pgen-0030116-b003] McLaren A (2003). Primordial germ cells in the mouse. Dev Biol.

[pgen-0030116-b004] Lyon MF (1961). Gene action in the X-chromosome of the mouse (Mus musculus L). Nature.

[pgen-0030116-b005] Takagi N, Sasaki M (1975). Preferential inactivation of the paternally derived X chromosome in the extraembryonic membranes of the mouse. Nature.

[pgen-0030116-b006] Heard E, Clerc P, Avner P (1997). X-chromosome inactivation in mammals. Annu Rev Genet.

[pgen-0030116-b007] Sun BK, Deaton AM, Lee JT (2006). A transient heterochromatic state in *Xist* preempts X inactivation choice without RNA stabilization. Mol Cell.

[pgen-0030116-b008] Brockdorff N, Ashworth A, Kay GF, McCabe VJ, Norris DP (1992). The product of the mouse *Xist* gene is a 15 kb inactive X-specific transcript containing no conserved ORF and is located in the nucleus. Cell.

[pgen-0030116-b009] Brown CJ, Hendrich BD, Rupert JL, Lafreniere RG, Xing Y (1992). The human XIST gene: Analysis of a 17 kb inactive X-specific RNA that contains conserved repeats and is highly localized within the nucleus. Cell.

[pgen-0030116-b010] Clemson CM, McNeil JA, Willard HF, Lawrence JB (1996). XIST RNA paints the inactive X chromosome at interphase: Evidence for a novel RNA involved in nuclear/chromosome structure. J Cell Biol.

[pgen-0030116-b011] Lee JT (2000). Disruption of imprinted X inactivation by parent-of-origin effects at *Tsix*. Cell.

[pgen-0030116-b012] Sado T, Wang Z, Sasaki H, Li E (2001). Regulation of imprinted X-chromosome inactivation in mice by *Tsix*. Development.

[pgen-0030116-b013] Epstein CJ (1969). Mammalian oocytes: X chromosome activity. Science.

[pgen-0030116-b014] Gartler SM, Andina R, Gant N (1975). Ontogeny of X chromosome inactivation in the female germ line. Exp Cell Res.

[pgen-0030116-b015] Andina RJ (1978). A study of X chromosome regulation during oogenesis in the mouse. Exp Cell Res.

[pgen-0030116-b016] Kratzer PG, Chapman VM (1981). X chromosome reactivation in oocytes of Mus caroli. Proc Natl Acad Sci U S A.

[pgen-0030116-b017] Monk M, McLaren A (1981). X-chromosome activity in foetal germ cells of the mouse. J Embryol Exp Morph.

[pgen-0030116-b018] Tam PPL, Zhou SX, Tan S-S (1994). X-chromosome activity of the mouse primordial germ cells revealed by the expression of an X-linked *lacZ* transgene. Development.

[pgen-0030116-b019] Nesterova TB, Mermoud JE, Hilton K, Pehrson J, Surani MA (2002). *Xist* expression and macroH2A1.2 localisation in mouse primordial and pluripotent embryonic germ cells. Differentiation.

[pgen-0030116-b020] Huynh KD, Lee JT (2003). Inheritance of a pre-inactivated paternal X chromosome in early mouse embryos. Nature.

[pgen-0030116-b021] Okamoto I, Otte AP, Allis CD, Reinberg D, Heard E (2004). Epigenetic dynamics of imprinted X inactivation during early mouse development. Science.

[pgen-0030116-b022] Okamoto I, Armaud D, Le Baccon P, Otte AP, Disteche CM (2005). Evidence for *de novo* imprinted X-chromosome inactivation independent of meiotic inactivation in mice. Nature.

[pgen-0030116-b023] Mak W, Nesterova TB, de Napoles M, Appanah R, Yamanaka S (2004). Reactivation of the paternal X chromosome in early mouse embryos. Science.

[pgen-0030116-b024] Grant SG, Chapman VM (1988). Mechanisms of X-chromosome regulation. Annu Rev Genet.

[pgen-0030116-b025] Surani MA, Hayashi K, Hajkova P (2007). Genetic and epigenetic regulators of pluripotency. Cell.

[pgen-0030116-b026] Gartler SM, Rivest M, Cole RE (1980). Cytological evidence for an inactive X chromosome in murine oogonia. Cytogenet Cell Genet.

[pgen-0030116-b027] Saitou M, Barton SC, Surani MA (2002). A molecular programme for the specification of germ cell fate in mice. Nature.

[pgen-0030116-b028] Sato M, Kimura T, Kurokawa K, Fujita Y, Abe K (2002). Identification of PGC7, a new gene expressed specifically in preimplantation embryos and germ cells. Mech Dev.

[pgen-0030116-b029] Ohinata Y, Payer B, O'Carroll D, Ancelin K, Ono Y (2005). Blimp1 is a critical determinant of the germ cell lineage in mice. Nature.

[pgen-0030116-b030] McLaren A, Lawson KA (2005). How is the mouse germ-cell lineage established?. Differentiation.

[pgen-0030116-b031] Campbell RE, Tour O, Palmer AE, Steinbach PA, Baird GS (2002). A monomeric red fluorescent protein. Proc Natl Acad Sci U S A.

[pgen-0030116-b032] Yoshimizu T, Sugiyama N, De Felice M, Yeom YI, Ohbo K (1999). Germline-specific expression of the Oct-4/green fluorescent protein (GFP) transgene in mice. Dev Growth Differ.

[pgen-0030116-b033] Ohbo K, Yoshida S, Ohmura M, Ohneda O, Ogawa T (2003). Identification and characterization of stem cells in prepubertal spermatogenesis in mice. Dev Biol.

[pgen-0030116-b034] Kikkawa Y, Miura I, Takahama S, Wakana S, Yamazaki Y (2001). Microsatellite database for MSM/Ms and JF1/Ms, *molossinus*-derived inbred strains. Mamm Genome.

[pgen-0030116-b035] Csankovszki G, Panning B, Bates B, Pehrson JR, Jaenisch R (1999). Conditional deletion of *Xist* disrupts histone macroH2A localization but not maintenance of X inactivation. Nat Genet.

[pgen-0030116-b036] Tam PPL, Snow MH (1981). Proliferation and migration of primordial germ cells during compensatory growth in mouse embryos. J Embryol Exp Morphol.

[pgen-0030116-b037] Takagi N, Yoshida MA, Sugawara O, Sasaki M (1983). Reversal of X-inactivation in female mouse somatic cells hybridization with murine teratocarcinoma stem cells in vitro. Cell.

[pgen-0030116-b038] Takagi N (1988). Requirement of mitosis for the reversal of X-inactivation in cell hybrids between murine embryonal carcinoma cells and normal female thymocytes. Exp Cell Res.

[pgen-0030116-b039] Sado T, Fenner MH, Tan S-S, Tam P, Shioda T (2000). X inactivation in the mouse embryo deficient for *Dnmt1*: Distinct effect of hypomethylation on imprinted and random X inactivation. Dev Biol.

[pgen-0030116-b040] Lewis A, Mitsuya K, Umlauf D, Smith P, Dean W (2004). Imprinting on distal Chromosome 7 in the placenta involves repressive histone methylation independent of DNA methylation. Nat Genet.

[pgen-0030116-b041] Seki Y, Hayashi K, Itoh K, Mizugaki M, Saitou M (2005). Extensive and orderly reprogramming of genome-wide chromatin modifications associated with specification and early development of germ cells in mice. Dev Biol.

[pgen-0030116-b042] Stack SM (1984). Heterochromatin, the synaptonemal complex and crossing over. J Cell Sci.

[pgen-0030116-b043] Sado T, Hoki Y, Sasaki H (2005). *Tsix* silences *Xist* through modification of chromatin structure. Dev Cell.

[pgen-0030116-b044] Lee EC, Yu D, Martinez de Velasco JM, Tessarollo L, Swing DA (2001). A highly efficient Escherichia coli-based chromosome engineering system adapted for recombinogenic targeting and subcloning of BAC DNA. Genomics.

[pgen-0030116-b045] Downs KM, Davies T (1993). Staging of gastrulating mouse embryos by morphological landmarks in the dissecting microscope. Development.

[pgen-0030116-b046] Kaufman MH (1995). The atlas of mouse development. Revised edition.

