# Supplementary material for: X Chromosome Reactivation Initiates in Nascent Primordial Germ Cells in Mice
Source: PLoS Genet. 2007 Jul 27;3(7):e116. doi: 10.1371/journal.pgen.0030116 (PMC1950944; doi:10.1371/journal.pgen.0030116)
Supplement: Figure S1 — Xist RNA signal is red, and Cot-1 signal is white. Oct4 immunofluorescence (green) was used to identify PGCs. Xist signal was not detected in any Oct4-positive cells at this stage. (195 KB PDF) [file pgen.0030116.sg001.pdf]

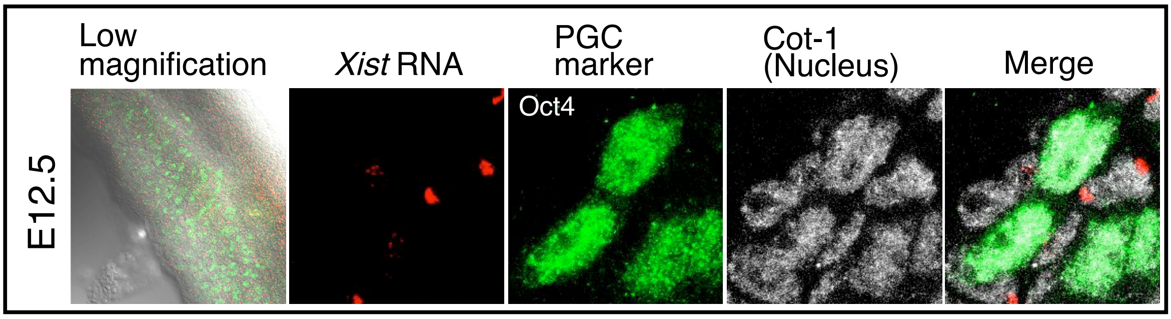

Figure S1. Whole-mount RNA FISH with *Xist* probe in E12.5 female PGCs. *Xist* RNA signal is red and Cot-1 signal is white. Oct4 immunofluorescence (green) was used to identify PGCs. *Xist* signal was not detected in any Oct4-positive cells at this stage.
