# Supplementary material for: X Chromosome Reactivation Initiates in Nascent Primordial Germ Cells in Mice
Source: PLoS Genet. 2007 Jul 27;3(7):e116. doi: 10.1371/journal.pgen.0030116 (PMC1950944; doi:10.1371/journal.pgen.0030116)
Supplement: Figure S2 — (A) Transgene construct expressing mRFP under the control of Blimp1 regulatory elements is shown (see Materials and Methods). (B) Transgene expression was observed under a fluorescence stereomicroscope. mRFP fluorescence was detected as a cluster of cells in the extraembryonic mesoderm (arrowhead) and in the visceral endoderm. (173 KB PDF) [file pgen.0030116.sg002.pdf]

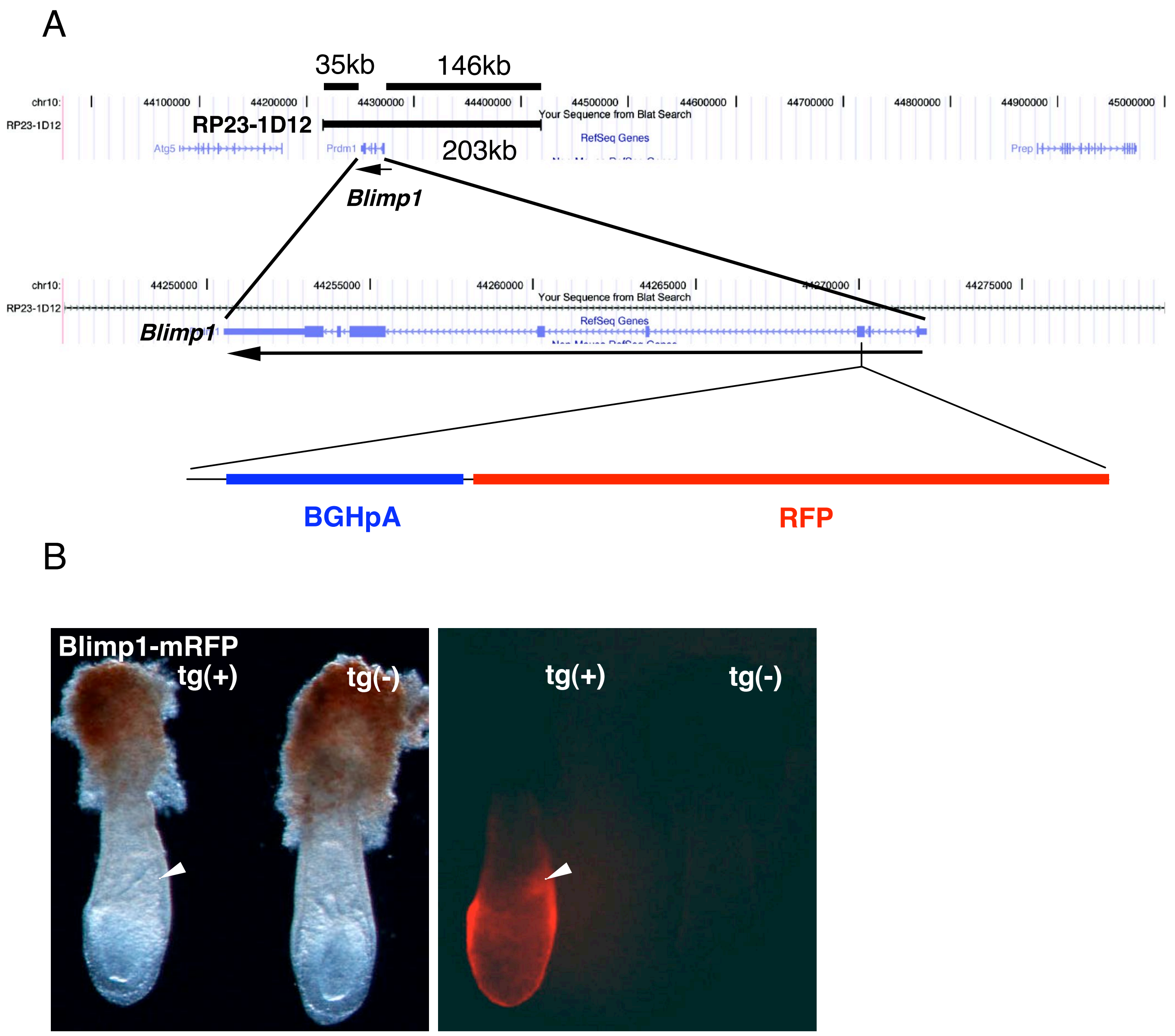

Figure S2. Generation of the Blimp1-mRFP transgenic mouse line. (A) Transgene construct expressing mRFP under the control of *Blimp1* regulatory elements (see Methods). (B) Transgene expression observed under a fluorescence stereomicroscope. mRFP fluoerescence was detected as a cluster in the extraembryonic mesoderm (arrowhead) and in the visceral endoderm.
