# Supplementary material for: X Chromosome Reactivation Initiates in Nascent Primordial Germ Cells in Mice
Source: PLoS Genet. 2007 Jul 27;3(7):e116. doi: 10.1371/journal.pgen.0030116 (PMC1950944; doi:10.1371/journal.pgen.0030116)
Supplement: Figure S3 — Tissues containing PGCs were isolated at E14.5 (A), E12.5 (C), E10.5 (E), E8.75 (G), and E7.75 (I). PGCs were identified as GFP-positive cells (B, D, F, H, and J) and picked manually (K, L). (311 KB PDF) [file pgen.0030116.sg003.pdf]

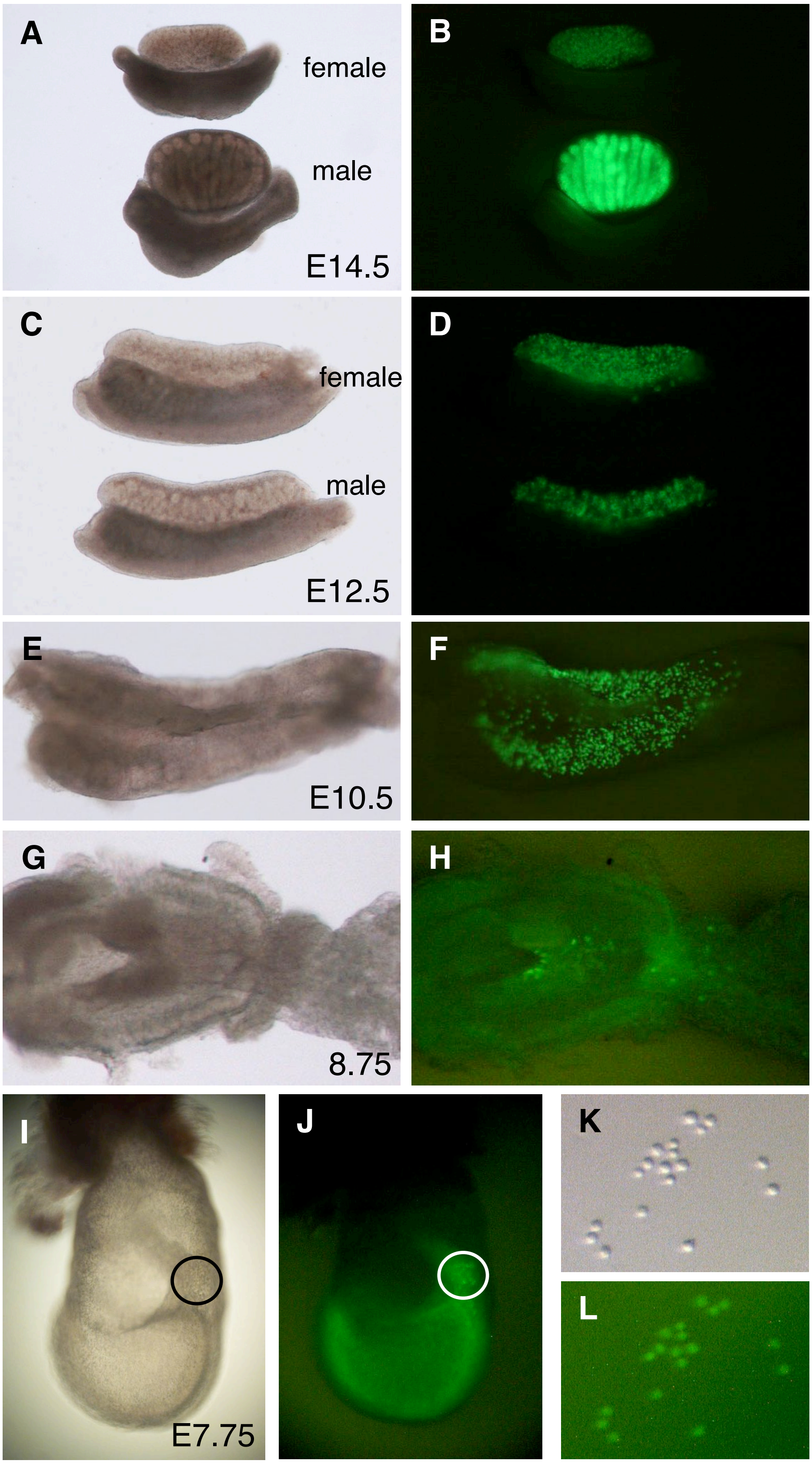

Figure S3. Oct4-GFP expression patterns during embryogenesis. Tissues containing PGCs were isolated at E14.5 (A), E12.5 (C), E10.5 (E), E8.75 (G), and E7.75 (I). PGCs were identified as GFP-positive cells (B, D, F H, J) and picked manually (K, L).
