# Supplementary material for: X Chromosome Reactivation Initiates in Nascent Primordial Germ Cells in Mice
Source: PLoS Genet. 2007 Jul 27;3(7):e116. doi: 10.1371/journal.pgen.0030116 (PMC1950944; doi:10.1371/journal.pgen.0030116)
Supplement: Figure S4 — E8.75 and E12.5 PGCs were isolated from hybrid embryos generated from Oct4-GFP × MSM/Ms matings, and oocytes were from superovulated hybrid females. MEFs and ES cells were used as controls. Asterisks indicate biallelic expression. (466 KB PDF) [file pgen.0030116.sg004.pdf]

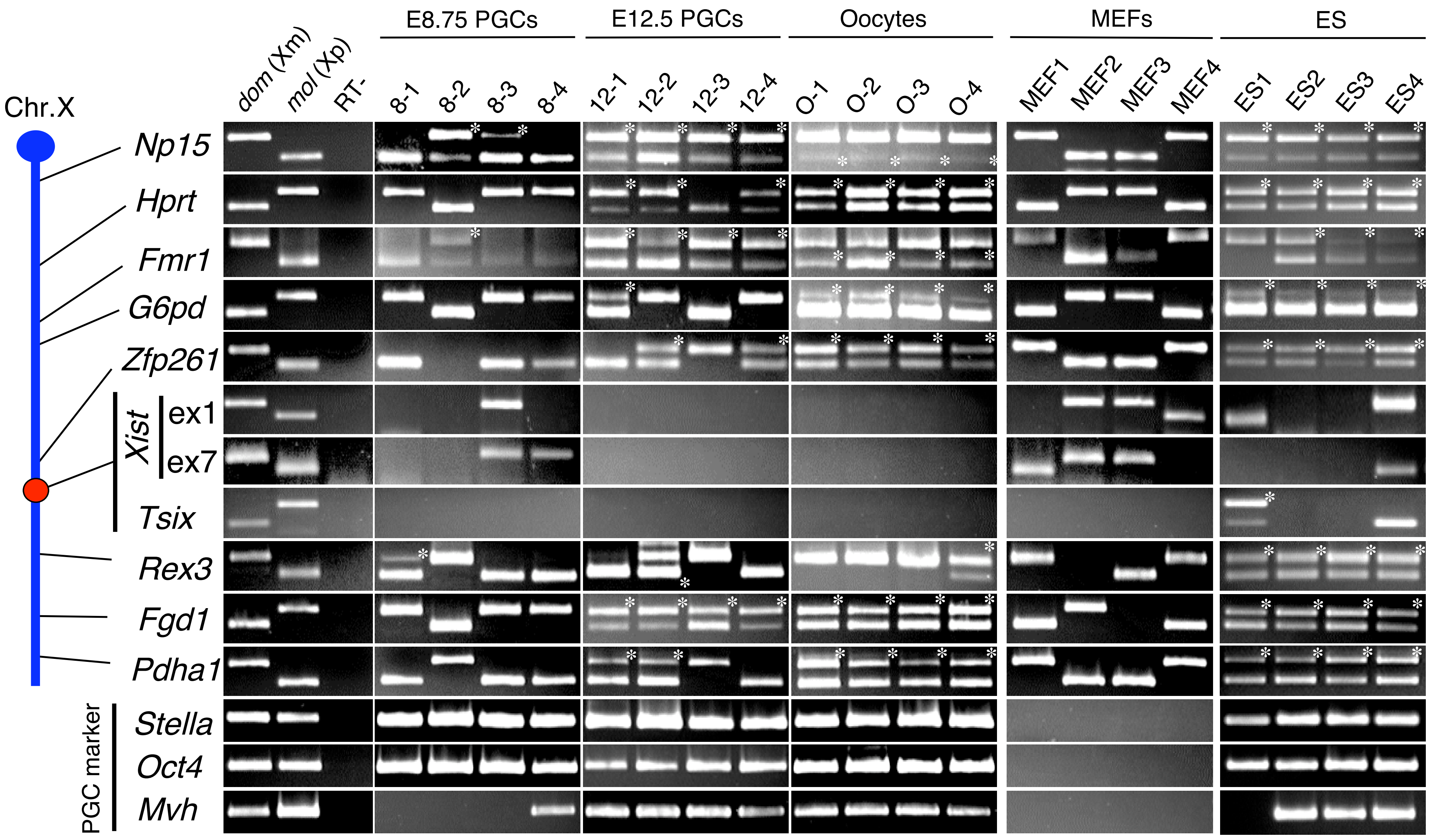

Figure. S4. Single-cell RT-PCR results of E8.75 PGCs, E12.5 PGCs, oocytes, MEFs, and ES cells. E8.75 and E12.5 PGCs were isolated from hybrid embryos generated from Oct4-GFP × MSM/Ms matings, and oocytes were from superovulated hybrid females. MEFs and ES cells were used as controls. Asterisks indicate biallelic expression.
