# Supplementary material for: X Chromosome Reactivation Initiates in Nascent Primordial Germ Cells in Mice
Source: PLoS Genet. 2007 Jul 27;3(7):e116. doi: 10.1371/journal.pgen.0030116 (PMC1950944; doi:10.1371/journal.pgen.0030116)
Supplement: Table S1 — (38 KB PDF) [file pgen.0030116.st001.pdf]

Table S1. Detailed expression pattern in single cells

+/- Xm (dom) expression; -/+ Xp (mol) expression; +/+ biallelic expression; (grey) not examined; +: expression was detected; -: expression was not detected

E7.75 PGCs

	7-1	7-2	7-3	7-4	7-5	7-6	7-7	7-8	7-9	7-10	7-11	7-12	7-13	7-14	7-15	7-16	7-17	7-18	7-19	7-20	7-21	monoallelic (B6/MSM)	biallelic	negative	total	% of biallelic (n)
Xist exon1	+/-	-	-	-	+/-	-/+	-	-	-	-	-	-	+/-	-/+	-	+/-	-	-	-	+/-	-	7 (5/2)	0	14	21	
Xist exon7	-	-/+	-/+	+/-	-	-	-	-	-	-	+/-	-/+	+/-	-	+/-	+/-	-	-	+/-	+/-	+/-	11 (8/3)	0	10	21	
Tsix				-	-	-	-	-	-	-	-	-	-	-	-	-	-	-	-/+	-	-	1 (0/1)	0	17	18	
Np15	+/+	+/-	+/+	-/+	-/+	+/-	+/-	+/-	+/-	+/-	-/+	+/-	-/+	+/-	-/+	-/+	+/-	+/-	-/+	-/+	-/+	19 (10/9)	2	0	21	9.5 (21)
Hprt	+/+	+/-	+/+	-/+	-/+	+/-	+/+	+/-	-	+/-	-/+	+/-	-/+	+/-	-/+	-/+	+/-	+/-	-/+	-/+	-/+	17 (8/9)	3	1	21	15.0 (20)
Fmr1	-/+	+/-	+/+	-/+	-	+/-	+/-	-	-	+/-	-	-	-/+	-	-/+	-	+/-	+/-	+/+	-/+	-/+	12 (6/6)	2	7	21	14.3 (14)
G6pd	-/+	+/-	+/+	-/+	-/+	+/-	+/-	+/-	+/-	+/-	-/+	+/-	-/+	+/-	-/+	-/+	-	+/-	-/+	-/+	-/+	19 (9/10)	1	1	21	5.0 (20)
Zfp261	+/+	+/-	+/+	-	-/+	-	+/+	-	-	-	-	-	-	+/-	-/+	-	-/+	-	-	-	-	5 (2/3)	3	13	21	37.5 (8)
Rex3	-/+	+/-	+/+	-/+	-/+	+/-	+/+	+/-	+/-	+/-	-/+	+/-	+/+	+/-	-/+	-/+	+/-	+/-	-/+	-/+	-/+	18 (9/9)	3	0	21	14.3 (21)
Fgd1	-/+	+/-	+/+	-/+	-/+	+/+	+/-	+/-	+/-	+/-	-/+	+/-	-/+	+/+	-/+	-/+	+/-	+/-	-/+	-/+	-/+	18 (8/10)	3	0	21	14.3 (21)
Pdha1	+/+	+/-	+/+	-/+	-/+	+/-	+/+	+/-	+/-	+/-	-/+	+/-	-/+	+/-	-/+	-/+	+/+	+/-	-/+	-/+	-/+	17 (8/9)	4	0	21	19.0 (21)
																						positive		negative		
Stella	+	+	+	+	+	+	+	+	+	+	+	+	+	+	+	+	+	+	+	+	+	21		0	21	
Oct4	+	+	+	+	+	+	+	+	+	+	+	+	+	+	+	+	+	+	+	+	+	21		0	21	
Mvh	-	-	-	-	-	+	+	+	-	+	-	-	+	-	+	+	-	-	+	-	+	9		12	21	

Xist(+) cells: 15/21 (71.4%)

E8.75 PGCs

	8-1	8-2	8-3	8-4	8-5	8-6	8-7	8-8	8-9	8-10	8-11	8-12	8-13	8-14	8-15	8-16	8-17	8-18	8-19	monoallelic (B6/MSM)	biallelic	negative	total	% of biallelic (n)
Xist exon1	-	+/-	-	-	-/+	-/+	-	-	-	-	-	-	+/-	+/-	-	-	+/-	-	-	6 (4/2)	0	13	19	
Xist exon7	-	+/-	-	-	-	-	-	-	-	-	-	-	+/-	-	+/-	+/-	-	-	-	4 (4/0)	0	15	19	
Tsix					-	-	-	-	-	-/+	-	-	-	-	-	-	-	-	-	1 (0/1)	0	14	15	
Np15	+/+	+/+	+/+	+/+	+/-	+/-	-/+	-/+	-/+	-/+	+/-	+/+	+/+	-/+	+/+	-/+	-/+	+/-	-/+	12 (4/8)	7	0	19	36.8 (19)
Hprt	-	-/+	+/-	+/-	+/-	+/-	-/+	-/+	-/+	-/+	+/-	+/-	-/+	-/+	-/+	-/+	-/+	+/-	-/+	18 (7/11)	0	1	19	0.0 (19)
Fmr1	-	-	-	-	+/-	+/-	-/+	+/-	-/+	-/+	+/-	+/+	-/+	-/+	-/+	-/+	-/+	+/-	-/+	14 (5/9)	1	4	19	6.7 (15)
G6pd	-	-/+	+/-	+/-	+/-	+/-	-/+	-/+	-/+	-/+	+/-	+/-	-/+	-/+	-/+	-/+	-/+	+/-	-/+	18 (7/11)	0	1	19	0.0 (19)
Zfp261	-/+	-/+	-	+/-	-	-	-/+	-/+	-/+	-	-	-	-/+	-	-/+	-/+	-/+	-	-	10 (1/9)	0	9	19	0.0 (19)
Rex3	+/+	-/+	+/-	+/-	+/-	+/-	+/+	-/+	-/+	-/+	+/-	+/-	-/+	-/+	-/+	-/+	-/+	+/-	-/+	17 (7/10)	2	0	19	10.5 (19)
Fgd1	+/+	+/+	+/-	+/-	+/-	+/-	-/+	-/+	-/+	-/+	+/-	+/-	-/+	-/+	-/+	-/+	-/+	+/-	-/+	17 (7/10)	2	0	19	10.5 (19)
Pdha1	-/+	-/+	+/-	+/-	+/-	+/-	-/+	-/+	-/+	-/+	+/-	+/-	-/+	-/+	-/+	-/+	-/+	+/-	-/+	19 (7/12)	0	0	19	0.0 (19)
																				positive		negative		
Stella	+	+	+	+	+	+	+	+	+	+	+	+	+	+	+	+	+	+	+	19		0	19	
Oct4	+	+	+	+	+	+	+	+	+	+	+	+	+	+	+	+	+	+	+	19		0	19	
Mvh	-	+	+	-	+	+	-	-	+	-	-	-	-	+	-	+	-	-	-	7		12	19	

Xist(+) cells: 8/19 (42.1%)

E10.5 PGCs

	10-1	10-2	10-3	10-4	10-5	10-6	10-7	10-8	10-9	10-10	10-11	10-12	10-13	10-14	10-15	10-16	10-17	10-18	10-19	monoallelic (B6/MSM)	biallelic	negative	total	% of biallelic (n)
Xist exon1	-	-	-	-	-	-	-	-	-	-	-	-	-	-	-	-	-	-	-	0	0	19	19	
Xist exon7	-	-	-	-	-	-	-	-	-	-	-	-	-	-	-	-	-	-	-	0	0	19	19	
Tsix								-	-	-	-	-	-	-	-	-	-	-	-/+	1 (0/1)	0	11	12	
Np15	+/+	+/+	-/+	+/+	+/+	+/+	+/+	-/+	+/+	+/+	+/+	+/+	+/+	+/+	+/+	+/+	+/+	+/+	+/+	2 (0/2)	17	0	19	89.5 (19)
Hprt	-	-/+	-/+	+/+	-/+	-/+	-/+	-/+	-/+	-/+	-/+	+/-	-/+	+/+	-/+	-/+	-/+	-/+	-/+	16 (1/15)	2	1	19	11.1 (18)
Fmr1	-	-	-	-	-	-	-	-/+	-/+	+/+	-/+	+/+	+/+	-/+	-/+	-/+	-/+	+/+	-/+	8 (0/8)	4	7	19	33.3 (12)
G6pd	-	-/+	-/+	-	-	-	-	-/+	-/+	-/+	-/+	+/-	-/+	-/+	-/+	-/+	-/+	-/+	-/+	14 (1/13)	0	5	19	0.0 (19)
Zfp261	+/+	-/+	-/+	+/+	+/+	-/+	-/+	-/+	-/+	-/+	-/+	+/+	-/+	-/+	-/+	+/+	+/+	-/+	-/+	13 (0/13)	6	0	19	31.6 (19)
Rex3	-/+	-/+	-/+	-/+	-/+	-/+	-/+	-/+	-/+	-/+	-/+	+/-	-/+	-/+	-/+	-/+	-/+	-/+	-/+	19 (1/18)	0	0	19	0.0 (19)
Fgd1	+/+	+/+	+/+	+/+	+/+	+/+	+/+	+/+	+/+	+/+	-/+	+/+	-/+	+/+	-/+	-/+	+/+	-/+	+/+	5 (0/5)	14	0	19	73.7 (19)
Pdha1	-	-/+	+/+	-	+/+	-/+	+/+	+/+	+/+	+/+	-/+	+/+	-/+	-/+	-/+	-/+	+/+	+/+	+/+	7 (0/7)	10	2	19	52.6 (19)
																				positive		negative		
Stella	+	+	+	+	+	+	+	+	+	+	+	+	+	+	+	+	+	+	+	19		0	19	
Oct4	+	+	+	+	+	+	+	+	+	+	+	+	+	+	+	+	+	+	+	19		0	19	
Mvh	+	+	+	+	+	+	+	+	+	+	+	+	+	+	+	+	+	+	+	19		0	19	

Xist(+) cells: 0/19 (0.0%)

E12.5 PGCs

	12-1	12-2	12-3	12-4	12-5	12-6	12-7	12-8	12-9	12-10	12-11	12-12	12-13	12-14	12-15	12-16	12-17	12-18	12-19	12-20	monoallelic (B6/MSM)	biallelic	negative	total	% of biallelic (n)
Xist exon1	-	-	-	-	-	-	-	-	+/-	-	-	-	-/+	-	-	-	-	-	-	-	2 (1/1)	0	18	20	
Xist exon7									-	-	-	-	-	-	-	-	-	-	-	-	0	0	12	12	
Tsix																	-	-	-	-	0	0	4	4	
Np15	+/+	+/+	+/+	+/+	+/+	+/+	+/+	+/+	+/+	+/+	+/+	+/+	+/+	+/+	+/+	+/+	+/+	+/+	+/+	+/+	0	20	0	20	100 (20)
Hprt	+/+	+/+	+/+	+/+	+/+	+/-	+/+	+/+	-/+	+/+	+/-	+/+	-/+	-/+	+/-	-/+	+/+	+/+	+/-	+/+	8 (4/4)	12	0	20	60.0 (20)
Fmr1	+/+	+/-	+/+	+/+	+/+	+/+	-	+/+	+/+	+/+	+/+	-/+	+/+	+/+	+/+	+/+	+/+	+/+	+/+	+/+	2 (1/1)	17	1	20	89.5 (19)
G6pd	-/+	+/-	-	-/+	-/+	+/-	-/+	-	-/+	-/+	+/-	-/+	-/+	-/+	-	+/+	+/+	-/+	+/-	-/+	15 (4/11)	2	3	20	11.8 (17)
Zfp261	+/+	+/-	-/+	+/+	+/+	+/-	-	+/+	-/+	-	+/-	-/+	-	+/+	+/+	-/+	-/+	+/+	+/-	+/+	9 (4/5)	8	3	20	47.1 (17)
Rex3	-/+	+/-	+/+	-/+	-/+	+/-	-/+	-/+	-/+	-/+	+/-	-/+	-/+	-/+	+/-	-/+	-/+	-/+	+/-	-/+	19 (5/14)	1	0	20	5.0 (20)
Fgd1	+/+	+/+	+/+	+/+	+/+	+/+	+/+	+/+	+/+	+/+	+/+	+/+	+/+	+/+	+/+	+/+	+/+	+/+	+/+	+/+	0	20	0	20	100 (20)
Pdha1	+/+	+/+	+/+	+/+	+/+	+/+	+/+	+/+	+/+	+/+	+/+	-/+	+/+	-/+	+/+	-/+	+/+	+/+	+/-	-/+	5 (1/4)	15	0	20	75.0 (20)
																					positive		negative		
Stella	+	+	+	+	+	+	+	+	+	+	+	+	+	+	+	+	+	+	+	+	20		0	20	
Oct4	+	+	+	+	+	+	+	+	+	+	+	+	+	+	+	+	+	+	+	+	20		0	20	
Mvh	+	+	+	+	+	+	+	+	+	+	+	+	+	+	+	+	+	+	+	+	20		0	20	

Xist(+) cells: 2/20 (10.0%)

E14.5 PGCs

	14-1	14-2	14-3	14-4	14-5	14-6	14-7	14-8	14-9	14-10	14-11	14-12	14-13	14-14	14-15	14-16	14-17	14-18	14-19	14-20	14-21	14-22	14-23	14-24	monoallelic (B6/MSM)	biallelic	negative	total	% of biallelic (n)
Xist exon1	-	-	-	-	-	-	-	-	-	-	-	-	-	-	+/-	-	-	+/-	-	-	-	-	-	-	2 (2/0)	0	22	24	
Xist exon7	-	-	-	-	-/+	-	-	-	-	-	-	-	-	-	-	-	-	-	-/+	-	-	-	-	-	2 (0/2)	0	22	24	
Tsix									-	-	-	-	-	-	-	-/+	-	-	-	-	-	-	-	-	1 (0/1)	0	15	16	
Np15	+/+	+/+	+/+	+/+	+/+	+/+	+/+	+/+	+/+	+/+	+/+	+/+	+/+	+/+	+/+	+/+	+/+	+/+	+/+	+/+	+/+	+/+	+/+	+/+	0	24	0	24	100 (24)
Hprt	+/+	+/+	+/+	+/+	+/+	+/+	+/+	+/+	+/+	+/+	+/+	+/+	+/+	+/+	+/+	+/+	+/+	+/+	+/+	+/+	+/+	+/+	+/+	+/+	0	24	0	24	100 (24)
Fmr1	-/+	+/+	+/+	+/+	+/+	-	+/+	+/+	+/+	+/+	+/+	+/+	+/+	-/+	+/+	+/+	+/+	+/+	+/+	-/+	+/+	+/+	+/+	-/+	4 (0/4)	19	1	24	82.6 (23)
G6pd	-	+/+	+/-	+/+	+/-	-/+	+/-	+/+	+/+	+/-	-/+	+/+	+/-	-	+/+	+/+	-/+	+/-	+/-	+/-	+/+	+/-	+/-	+/-	14 (11/3)	8	2	24	9.1 (22)
Zfp261	+/+	+/+	+/+	+/+	+/-	-/+	+/+	+/+	+/+	+/+	+/+	+/+	+/+	+/+	+/+	+/+	+/+	+/+	+/+	+/+	+/+	+/+	+/+	+/+	2 (1/1)	22	0	24	91.7 (24)
Rex3	-/+	+/+	+/-	+/-	+/-	+/-	+/-	-/+	+/+	-/+	-/+	+/+	-/+	+/+	-/+	-/+	-/+	+/+	-/+	+/-	-/+	+/-	+/+	+/-	18 (8/10)	6	0	24	25.0 (24)
Fgd1	+/+	+/+	-	+/+	+/-	-	-	+/+	-/+	+/+	+/+	+/+	-	-/+	+/+	-	-	-/+	-	+/-	-/+	-	-	-/+	7 (2/5)	8	9	24	53.3 (15)
Pdha1	-	+/+	+/+	-/+	+/+	-/+	+/+	-	+/+	-	+/+	-	-/+	+/-	+/-	-/+	+/-	-/+	-	-/+	+/+	+/-	+/-	-	11 (5/6)	7	6	24	38.9 (18)
																									positive		negative		
Stella	-	+	-	+	+	+	-	-	+	-	-	-	-	-	-	+	+	-	-	-	-	-	+	-	8		16	24	
Oct4	-	+	-	+	-	-	-	-	-	-	-	-	+	-	-	-	-	-	-	-	-	-	-	+	4		20	24	
Mvh	+	+	+	+	+	+	+	+	+	+	+	+	+	+	+	+	+	+	+	+	+	+	+	+	24		0	24	

Xist(+) cells: 4/24 (16.7%)

Oocytes

	O-1	O-2	O-3	O-4	O-5	O-6	O-7	O-8	O-9	O-10	O-11	O-12	O-13	O-14	monoallelic (B6/MSM)	biallelic	negative	total	% of biallelic (n)
Xist exon1	-	-	-	-	-	-	-	-	-	-	-	-	-	-	0	0	14	14	
Xist exon7	-	-	-	-	-	-	-	-	-	-	-	-	-	-	0	0	14	14	
Tsix	-	-	-	-	-	-	-	-	-	-	+/-	-	-	-	1 (1/0)	0	13	14	
Np15	+/+	+/+	+/+	+/+	+/+	+/+	+/+	+/+	+/+	+/+	+/+	+/+	+/+	+/+	0	14	0	14	100 (14)
Hprt	+/+	+/+	+/+	+/+	+/+	+/+	+/+	+/+	+/+	+/+	+/+	+/+	+/+	+/+	0	14	0	14	100 (14)
Fmr1	+/+	+/+	+/+	+/+	+/+	+/+	+/+	+/+	+/+	+/+	+/+	+/+	+/+	+/+	0	14	0	14	100 (14)
G6pd	+/+	+/+	+/+	+/+	+/+	+/+	+/+	+/+	+/+	+/+	+/+	+/+	+/+	+/+	0	14	0	14	100 (14)
Zfp261	+/+	+/+	+/+	+/+	+/+	+/+	+/+	+/+	+/+	+/+	+/+	+/+	+/+	+/+	0	14	0	14	100 (14)
Rex3	+/+	+/-	+/-	+/-	+/-	+/-	+/-	+/-	+/-	+/-	+/-	-	+/-	+/+	11 (11/0)	2	1	14	15.4 (13)
Fgd1	+/+	+/+	+/+	+/+	+/+	+/+	+/+	+/+	+/+	+/+	+/+	+/+	+/+	+/+	0	14	0	14	100 (14)
Pdha1	+/+	+/+	+/+	+/+	+/+	+/+	+/+	+/+	+/+	+/+	+/+	+/+	+/+	+/+	0	14	0	14	100 (14)
															positive		negative		
Stella	+	+	+	+	+	+	+	+	+	+	+	+	+	+	14		0	14	
Oct4	+	+	+	+	+	+	+	+	+	+	+	+	+	+	14		0	14	
Mvh	+	+	+	+	+	+	+	+	+	+	+	+	+	+	14		0	14	

Xist(+) cells: 0/14 (0.0%)

Embryonic fibroblast

	MEF-1	MEF-2	MEF-3	MEF-4	MEF-5	MEF-6	MEF-7	MEF-8	MEF-9	MEF-10	MEF-11	MEF-12	MEF-13	MEF-14	MEF-15	MEF-16	MEF-17	MEF-18	MEF-19	MEF-20	MEF-21	MEF-22	MEF-23	MEF-24	MEF-25	MEF-26	monoallelic (B6/MSM)	biallelic	negative	total	% of biallelic (n)
Xist exon1	+/-	+/-	-/+	-	-/+	+/-	-/+	-	+/-	-	-	-	+/-	-/+	-	-	-/+	-/+	-	-	-	-	+/-	+/-	-/+	-	14 (7/7)	0	12	26	
Xist exon7	+/-	+/-	-	-	-/+	-	-/+	-	+/-	-	-/+	+/-	+/-	-/+	+/-	-	-	-	-	-	-/+	+/-	+/-	+/-	-	-	14 (9/5)	0	12	26	
Tsix											-	-	-	-	-	-	-	-	-	-	-	-	-	-	-	-	0	0	16	16	
Np15	-/+	-/+	+/-	-/+	+/-	-/+	+/-	+/-	-/+	+/-	+/-	-/+	-/+	+/-	-/+	+/-	+/-	+/-	-/+	+/-	+/-	-/+	-/+	-/+	+/-	-/+	26 (13/13)	0	0	26	0.0 (26)
Hprt	-/+	-/+	+/-	-/+	+/-	-/+	-	+/-	-/+	+/-	+/-	-/+	-/+	+/-	-/+	+/-	+/-	-	-/+	+/-	+/-	-/+	-/+	-/+	+/-	-/+	24 (11/13)	0	2	26	0.0 (24)
Fmr1	-/+	-/+	+/-	-/+	+/-	-	-	+/-	-/+	-	+/-	-/+	-/+	+/-	-/+	-	+/-	+/-	-/+	-	+/-	-/+	-	-/+	+/-	-/+	20 (9/11)	0	6	26	0.0 (20)
G6pd	-/+	-/+	+/-	-/+	+/-	-/+	-	+/-	-/+	+/-	+/-	-/+	-/+	+/-	-/+	+/-	+/-	+/-	-/+	+/-	+/-	-/+	-/+	-/+	+/-	-/+	25 (12/13)	0	1	26	0.0 (25)
Zfp261	-	-	-	-	+/-	-/+	+/-	+/-	-/+	+/-	+/-	-/+	-/+	+/+	-/+	-	+/-	+/-	-/+	+/-	+/-	-/+	-/+	-/+	+/-	-/+	20 (10/8)	1	5	26	4.8 (21)
Rex3	-/+	-	-	-	-	-	-	+/-	-	-	+/-	-	-	-	-/+	-	-	-	-	-	-	-	-	-/+	+/-	-/+	7 (3/4)	0	19	26	0.0 (7)
Fgd1	-/+	-/+	+/-	-/+	+/-	-/+	+/-	+/-	-/+	+/-	+/-	-/+	-/+	+/-	-/+	+/-	+/-	+/-	-/+	+/-	+/-	-/+	-/+	-/+	+/-	-/+	26 (13/13)	0	0	26	0.0 (26)
Pdha1	-/+	-/+	+/-	-/+	+/-	-/+	+/-	+/-	-/+	+/-	+/-	-/+	-/+	+/-	-/+	+/-	+/-	+/-	-/+	+/-	+/-	-/+	-/+	-/+	+/-	-/+	26 (13/13)	0	0	26	0.0 (26)
																											positive		negative		
Stella	-	-	-	-	-	-	-	-	-	-	-	-	-	-	-	-	-	-	-	+	-	-	-	-	-	-	1		25	26	
Oct4	-	-	-	-	-	-	-	-	-	-	-	-	-	-	-	-	-	-	-	-	-	-	-	-	-	-	0		26	26	
Mvh	-	-	-	-	-	-	-	-	-	-	-	-	-	-	-	-	-	-	-	-	-	-	-	-	-	-	0		26	26	

Xist(+) cells: 19/26 (73.1%)

ES cells

	ES-1	ES-2	ES-3	ES-4	ES-5	ES-6	ES-7	ES-8	ES-9	ES-10	monoallelic (B6/MSM)	biallelic	negative	total	% of biallelic (n)
Xist exon1	-	-	+/-	-	+/-	-	-	+/-	-	-/+	4 (3/1)	0	6	10	
Xist exon7	-	-	-	-	-	-	-	-/+	-	-/+	2 (0/2)	0	8	10	
Tsix	-/+	-/+	+/+	-	-	-	+/+	+/-	-	-	3 (1/2)	2	5	10	
Np15	+/+	+/+	+/+	+/+	+/+	+/+	+/+	+/+	+/+	+/+	0	10	0	10	100 (10)
Hprt	+/+	+/+	+/+	+/+	+/+	+/+	+/+	+/+	+/+	+/+	0	10	0	10	100 (10)
Fmr1	+/+	-	+/-	+/+	+/+	+/+	+/+	+/+	+/+	+/+	1 (1/0)	8	1	10	88.9 (9)
G6pd	+/+	+/+	+/+	+/+	+/+	+/+	+/+	+/+	+/+	+/+	0	10	0	10	100 (10)
Zfp261	+/+	-/+	+/+	+/+	+/+	+/+	+/+	+/+	-/+	+/+	2 (0/2)	8	0	10	80.0 (10)
Rex3	+/+	+/+	+/+	+/+	+/+	+/+	+/+	+/+	+/+	+/+	0	10	0	10	100 (10)
Fgd1	+/+	+/+	+/+	+/+	+/+	+/+	+/+	+/+	+/+	+/+	0	10	0	10	100 (10)
Pdha1	+/+	+/+	+/+	+/+	+/+	+/+	+/+	+/+	+/+	+/+	0	10	0	10	100 (10)
											positive		negative		
Stella	+	+	+	+	+	+	+	+	+	+	10		0	10	
Oct4	+	+	+	+	+	+	+	+	+	+	10		0	10	
Mvh	+	+	-	+	+	+	+	+	+	-	8		0	10	

Xist(+) cells: 4/10 (40.0%)
