# Supplementary material for: X Chromosome Reactivation Initiates in Nascent Primordial Germ Cells in Mice
Source: PLoS Genet. 2007 Jul 27;3(7):e116. doi: 10.1371/journal.pgen.0030116 (PMC1950944; doi:10.1371/journal.pgen.0030116)
Supplement: Table S2 — (16 KB PDF) [file pgen.0030116.st002.pdf]

Table S2. List of primer pairs and restriction enzymes used in single-cell RT-PCR

	forward	reverse	PCR product size	restriction enzyme	restriction fragments
Xist exon1	5'-CTAAAACTCAGCCCGTTCCA-3'	5'-GCAACCCCAGCAATAGTCAT-3'	217bp (dom)<br>225bp (mol)	SpeI	217bp (dom)<br>117, 48bp (mol)
Xist exon 7	5'-GCCCAGGTCACATTATGGTT-3'	5'-CTCCAATTTCTGGGCTCAAG-3'	232bp (dom)<br>241bp (mol)	SacI	232bp (dom)<br>197, 44bp(mol)
Tsix	5'-TGGGTCATTGGCATCTTAGTC-3'	5'-CCCAGGGTGTCTGATCTCTT-3'	250bp	Alw26I	159, 91bp (dom)<br>250bp (mol)
Np15	5'-CTCCGCAGTACAAGCTGTCA-3'	5'-CCATGAAAGTCAGGGTTCTTC-3'	237bp	PvuII	237bp (dom)<br>148, 89bp (mol)
Hprt	5'-TGTGGCCATCTGCCTAGTAA-3'	5'-CAGCCAACACTGCTGAAACA-3'	399bp	HinfI	314, 85bp (dom)<br>399bp (mol)
Fmr1	5'-CTTAACACTTCAGGGCAGGA-3'	5'-CTTCCCTGAACTCTGCATCC-3'	379bp	RsaI	379bp (dom)<br>273, 106bp (mol)
G6pd	5'-TTCTAGTTCCTGGGCTTGGA-3'	5'-TTAATGGCAGGGTTGGGATA-3'	568bp	DraI	473, 95bp (dom)<br>568bp (mol)
Zfp261	5'-GAGCAGACTCCTATTGTGCAG-3'	5'-AGGGAGGCAGCTTACAGAGG-3'	125bp	DdeI	125bp (dom)<br>99, 26bp (mol)
Rex3	5'-TAGATGGGACCTGATGCAGA-3'	5'-GAAGCTGGTAACAGGGAGAGA-3'	296bp	BsrGI	241, 55bp (dom)<br>183, 58, 55bp (mol)
Fgd1	5'-TCACACAAAGCCACCTAAGC-3'	5'-ATTGACTGCATTGGGAGTGG-3'	301bp	HhaI	243, 58bp (dom)<br>301bp (mol)
Pdha1	5'-TTCCAGCGATATGCTGACTTT-3'	5'-TGGCAAGGCATGAAGTGATA-3'	481bp	TaqI	481bp (dom)<br>351, 130bp (mol)
Stella	5'-GAGCCATTCAGATGTCTC-3'	5'-CACATAAATCTGGATCGTTGTG -3'<br>5'-CACATAAATCCGtATCTTTGTG-3'	238bp		
Oct4	5'-GTTCAGCCAGACCACCATCT-3'	5'-TGGGAAAGGTGTCCCTGTAG-3'	385bp		
Mvh	5'-GCCAGAGGGCTTGATATTGA-3'	5'-CAACTGGATTGGGAGCTTGT-3'	370bp		
